# PD102 Evaluating The Clinical Effectiveness Of Portable Ultrasonic Scalpels For Urological Surgery

**DOI:** 10.1017/S0266462324003477

**Published:** 2025-01-07

**Authors:** Juntao Yan, Yi Yang, Ying Tao, Yingyao Chen

## Abstract

**Introduction:**

Ultrasonic scalpels are widely used in urological surgery. Although portable ultrasonic scalpels are convenient to use and install, the existing evidence on their safety and effectiveness is scarce. This study aimed to compare the safety and effectiveness of portable ultrasonic scalpels in urological surgery with traditional ultrasonic scalpels to aid clinical decision-making.

**Methods:**

A multicenter, prospective, non-randomized controlled trial was conducted from February to August 2023 in three tertiary hospitals in China. The intervention group included 90 prospectively enrolled patients undergoing urological surgery during the same period of hospitalization: 45 with portable ultrasonic scalpels and 45 with traditional scalpels. Demographic and clinical data of patients in the study were collected. Data on quality of life were obtained using the EuroQol EQ-5D-5L scale preoperatively, at discharge, and one month and three months after surgery. Descriptive analysis and a generalized linear model were used in the data analysis.

**Results:**

A total of 82 patients were included in the study: 39 in the intervention group and 43 in the control group. The average hospital stay and intraoperative and postoperative blood loss in the intervention group were lower than in the control group (p>0.05). From baseline to discharge, the decrease in quality-adjusted life-years (QALYs) in the intervention group was smaller (–0.134 versus –0.287; p<0.05) than in the control group. During the follow-up period, there were no significant differences in the changes in QALYs between the two groups. The decline in QALYs was significantly influenced by variables such as intraoperative blood loss and surgical site.

**Conclusions:**

There were no significant differences in baseline characteristics or changes in QALYs between the intervention and control groups. Portable ultrasonic scalpels in urological surgery may be as equally effective as traditional scalpels with respect to clinical outcomes, with additional benefits in reducing QALY decline at discharge. Further research with large samples and long-term follow-up should be conducted.

